# Multiorgan Involvement in SARS-CoV-2 Infection: The Role of the Radiologist from Head to Toe

**DOI:** 10.3390/diagnostics12051188

**Published:** 2022-05-10

**Authors:** Davide Ippolito, Federica Vernuccio, Cesare Maino, Roberto Cannella, Teresa Giandola, Maria Ragusi, Vittorio Bigiogera, Carlo Capodaglio, Sandro Sironi

**Affiliations:** 1 Department of Diagnostic Radiology, San Gerardo Hospital, Via Pergolesi 33, 20900 Monza, MB, Italy; davide.atena@tiscali.it (D.I.); mainocesare@gmail.com (C.M.); teresagiandola1990@gmail.com (T.G.); maria.ragusi@gmail.com (M.R.); v.bigiogera@campus.unimib.it (V.B.); carloalberto.capodaglio@gmail.com (C.C.); 2 School of Medicine, University of Milano-Bicocca, Via Cadore 48, 20900 Monza, MB, Italy; davelegolas@gmail.com; 3 Department of Radiology, University Hospital of Padova, Via Nicolò Giustiniani, 2, 35128 Padova, PD, Italy; 4 Department of Biomedicine, Neuroscience and Advanced Diagnostics, University of Palermo, Via del Vespro, 129, 90127 Palermo, PA, Italy; rob.cannella89@gmail.com; 5 Department of Health Promotion, Mother and Child Care, Internal Medicine and Medical Specialties (PROMISE), University of Palermo, Via del Vespro, 129, 90127 Palermo, PA, Italy; 6 Department of Diagnostic Radiology, H Papa Giovanni XXIII, Piazza OMS 1, 24127 Bergamo, BG, Italy

**Keywords:** infections, coronavirus, radiography, tomography, X-ray computed

## Abstract

Radiology plays a crucial role for the diagnosis and management of COVID-19 patients during the different stages of the disease, allowing for early detection of manifestations and complications of COVID-19 in the different organs. Lungs are the most common organs involved by SARS-CoV-2 and chest computed tomography (CT) represents a reliable imaging-based tool in acute, subacute, and chronic settings for diagnosis, prognosis, and management of lung disease and the evaluation of acute and chronic complications. Cardiac involvement can be evaluated by using cardiac computed tomography angiography (CCTA), considered as the best choice to solve the differential diagnosis between the most common cardiac conditions: acute coronary syndrome, myocarditis, and cardiac dysrhythmia. By using compressive ultrasound it’s possible to study the peripheral arteries and veins and to exclude the deep vein thrombosis, directly linked to the onset of pulmonary embolism. Moreover, CT and especially MRI can help to evaluate the gastrointestinal involvement and assess hepatic function, pancreas involvement, and exclude causes of lymphocytopenia, thrombocytopenia, and leukopenia, typical of COVID-19 patients. Finally, radiology plays a crucial role in the early identification of renal damage in COVID-19 patients, by using both CT and US. This narrative review aims to provide a comprehensive radiological analysis of commonly involved organs in patients with COVID-19 disease.

## 1. Introduction

Since SARS-CoV-2 has been declared a pandemic by the World Health Organization in March 2020, global attention and strength were shifted to know, understand, and fight the virus. The effort was unanimous in every sector of science, technology, and health care institution, enforcing the medical treatment and organization, reconverting the workforce, developing Artificial Intelligence (AI) tools to help medical decisions, collaborating to carry on the expertise in every field of normal life, enhancing the best management possible of COVID-19 outbreak.

In this setting, considering the wide manifestation of SARS-CoV-2 in the whole body, diagnostic imaging plays a crucial role in the early detection of manifestations and complications of COVID-19 [1].

The *primum movens* pathological factor responsible for COVID-19 disease is the angiotensin-converting enzyme 2 (ACE2) receptor, widely expressed in different mammalian cells and human organs, including lung, cardiac, brain, and renal tissues. The viral protein of SARS-CoV-2 binds to the ACE2 receptor and enters different cells, causing various and different clinical scenarios [2].

It is now known that one hallmark of the disease is acute respiratory distress syndrome (ARDS), which was the driving focus of research and treatment, because it is the most common and severe feature, accounting for about 20% of recovered patients during the first wave of pandemic [3]. Chest X-rays and Computed Tomography (CT) are sensitive and effective techniques to identify, follow, and quantify the lung involvement and to drive the clinical decision, such as the need for mechanical ventilation and intensive care unit (ICU) recovery, and forecast or confirm doubt RT-PCR test [4,5].

The expression of ACE2 receptor in cardiomyocytes and the systemic inflammatory response syndrome can lead to cardiovascular involvement, including acute coronary syndrome, myocarditis, cardiac dysrhythmia [6], and thrombotic manifestations [7], which can be diagnosticated with CT and ultrasound (US).

Moreover, a growing incidence of a wide range of neurological manifestations confirms the hypothesis of COVID-19 neuro-invasion, and Magnetic Resonance Imaging (MRI) should be considered the reference standard in these scenarios [8].

Finally, COVID-19 infection may cause involvement of abdominal organs due to direct viral infection, thromboembolic complications, or indirect drug-induced complications [9,10,11]. In these settings, US and CT should be performed to support the final diagnosis and decide the best management.

**Figure 1:**
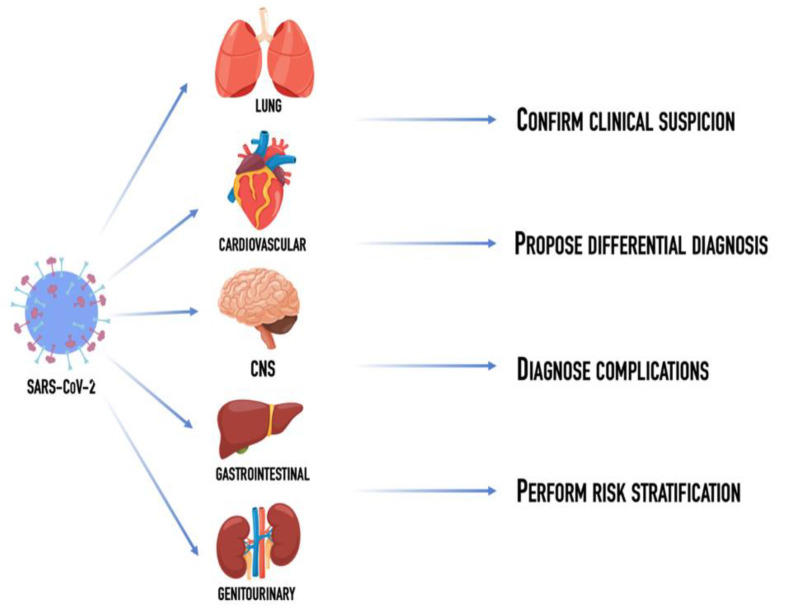
Schematic representation of the role of diagnostic radiology: confirm clinical suspicion in case of non-diagnostic polymerase chain reaction test, help solving the differential diagnosis, identify and follow the progression of possible complications and, finally, perform a risk stratification of patients affected with SARS-CoV-2.

On these bases, this narrative review aims to illustrate pearls and pitfalls of radiological techniques, to suggest how to investigate the different manifestations of the disease, to depict common and uncommon whole-body radiological findings (Figure 1).

## 2. Lung Involvement

### 2.1. The Usefulness of Chest CT

Chest CT represents a reliable imaging-based tool in acute, subacute, and chronic settings for diagnosis, prognosis, and management of lung disease and the evaluation of acute and chronic complications of patients with COVID-19 infection [4,5].

According to the European Society of Radiology and European Society of Thoracic Imaging (ESR/ESTI) advice paper and recommendations of the French Society of Thoracic Imaging, unenhanced chest CT during the first wave of the pandemic was indicated for patients presenting with dyspnea, polypnea, or desaturation, pending RT-PCR results [12].

A recent meta-analysis, reported a chest CT pooled sensitivity of 94.6% and a pooled specificity of 46% in the detection of COVID-19; however, 10.6% of symptomatic patients with RT-PCR test-proved COVID 19 have normal chest CT findings suggesting a significant number of false-negative [13].

CT examination could also lead to a high number of false positives due to overlapping imaging features with a wide spectrum of other diseases, such as other viral pneumonia [14].

Even if CT is not the reference standard for the diagnosis of COVID-19, the Fleischner Society published a consensus statement on the use of chest imaging, underlying that it is not indicated as a screening test in asymptomatic patients or patients with mild respiratory symptoms [15].

On the other hand, chest CT represents a useful technique along the course of the disease, especially to stratify the severity of lung involvement and to predict outcomes in COVID-19 helping to a proper triaging of patients and allocation of resources [16].

### 2.2. Typical CT Findings

The typical pulmonary histologic damage in COVID-19 pneumonia includes acute and organizing diffuse alveolar damage [17].

The primary and most frequent findings on CT, reported in more than 70% of RT-PCR test-proven COVID-19 cases, include ground-glass opacities (GGOs), crazy paving appearance, air space consolidations, broncho-vascular thickening, and vascular enlargement within pneumonia areas [18,19]. Among them, the most typical CT features of COVID-19 pneumonia are bilateral and multifocal GGOs which are predominantly distributed in the peripheral, posterior, and lower zones [20].

According to literature, in the early phase of the disease, the GGOs may present as a unifocal lesion, most commonly located in the inferior lobe of the right lung [21].

Pulmonary artery branch dilatation has been proposed as an early predictor of lung impairment; indeed, it usually occurs in locations where lung abnormalities will likely develop in the short term [22].

Chest CT anomalies with a low incidence (<10%) in patients with COVID-19 infection include pleural effusion (5.2%), lymphadenopathy (5.1%), tree-in-bud sign (4.1%), central lesion distribution (3.6%), pericardial effusion (2.7%), and cavitating lung lesions (0.7%) [17].

### 2.3. CT Findings Evolution

Knowledge of the natural temporal evolution of lung abnormalities in patients with COVID-19 pneumonia is necessary for the radiologist to better evaluate the prognosis and the risk of complications [5,12,13,16,17,18,19].

**Figure 2:**
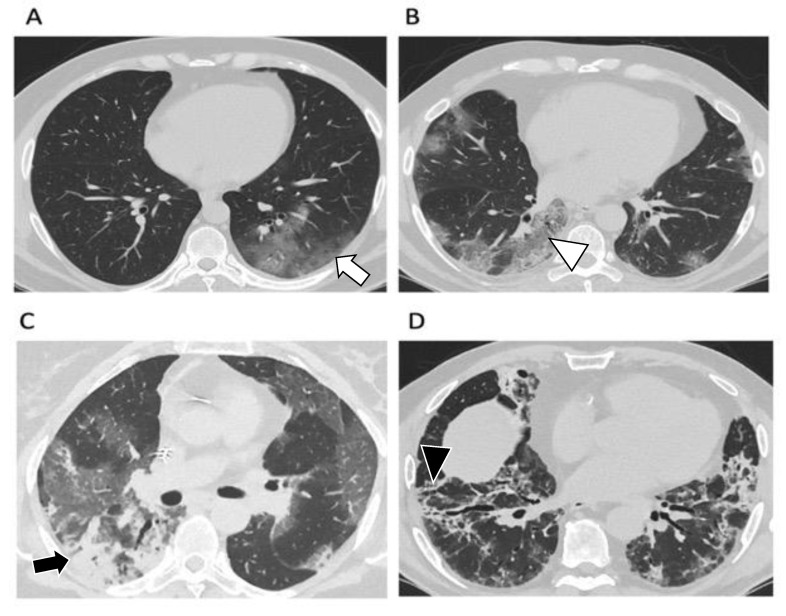
Axial chest CT images with windowing and leveling for the evaluation of lung parenchyma in patients affected by SARS-CoV-2 according to the different stages of the disease. (**A**). Early or initial stage (0–4 days): normal CT or sporadic ground-glass opacities (white arrow). (**B**). Progressive stage (5–8 days): ground-glass opacities are widely distributed, and crazy paving appearance can be evident (white arrowhead). (**C**). Peak stage (9–13 days): lung consolidations (black arrow) appear nearby the ground-glass opacities. (**D**). Absorption stage (>14 days): consolidations and ground-glass opacities slowly disappear while fibrotic findings can be evident, especially in the peripheral and lower zones (black arrowhead).

Four stages on CT have been described [23,24]: early/initial (0–4 days) characterized by normal CT or GGO only; progressive (5–8 days) with increased GGOs and crazy paving appearance; peak (9–13 days) characterized by lung consolidation and absorption stage (>14 days) with the appearance of fibrotic changes (Figure 2).

Lung damage is maximal at around day 10 and then generally decreases progressively in size and attenuation value [25].

Lung abnormalities on CT may persist beyond one month in up to 98% of patients, particularly in those with initial severe lung disease at baseline [24]. Nevertheless, the long-term sequelae of COVID-19 and the associated lung abnormalities remain uncertain.

### 2.4. Disease Severity

The density of pulmonary lesions, pleural effusion, early architectural distortion, bronchial dilatations, and consolidations in upper lobes on initial CT seem to be associated with poor outcomes [26,27,28].

Several studies have proposed CT-based semi-quantitative scores to evaluate the extent of lung involvement in COVID-19 pneumonia [28,29,30], to predict outcome and choose the correct management.

The Radiological Society of North America (RSNA) has published a Chest CT classification system for reporting COVID-19 pneumonia, based on lung involvement percentage by scoring the percentage of each lobe involvement individually [31]. The cut-off value for identifying severe cases of COVID-19 of CT score was reported as “17”, with 80.0% and 82.8% sensitivity and specificity, respectively [31].

Moreover, the use of a semi-automated CT algorithm for segmentation and quantification of the ventilated lung correlates with disease severity, laboratory parameters, and outcome, as well as the need for invasive ventilation [32].

The RSNA has provided a guidance document for reporting chest CT imaging findings in typical, indeterminate, atypical, and negative (Table 1) [33].

A study evaluating the RSNA chest CT classification system for COVID-19 against RT-PCR results found moderate to a substantial inter-observer agreement [29].

### 2.5. Complications

In patients with clinical worsening, chest imaging is suggested also to progression or complications. About 15–30% of hospitalized patients progress to ARDS, which is the main cause of mortality [34].

It has been reported that COVID-19 related ARDS can develop 8–12 days after symptoms onset [34] which is longer than the 1-week onset limit according to Berlin definition.

Barotrauma, including pneumothorax, pneumomediastinum, and pneumopericardium, have been reported in intubated patients [31]. Longer hospital length of stay and younger age were associated with a higher incidence of barotrauma events [34].

In COVID-19 patients there is an exacerbated systemic inflammatory response leading to a hypercoagulability state with a marked increase of D-dimer serum level, that may result in pulmonary embolism in about 17% and 35% of cases with an average time to diagnosis of around 16 days after symptoms onset [35].

### 2.6. The Usefulness of Chest X-ray

Although CT has good sensitivity and specificity for the diagnosis of COVID-19 pneumonia, the adoption of chest X-ray (CXR) during pandemic peaks and slumps may help to reduce the spread of the virus, avoid the overload of CT room and have a quick glaze of lung involvement. Nevertheless, only a few studies assessed the role of CXR for COVID-19 pneumonia [36].

Similar to chest CT, the most common and typical CXR findings are GGOs and consolidations, especially with a bilateral, lower, and peripheral zones distribution, with an overall sensitivity of 67% [37].

During pandemics peaks, CXR may help at admission in the emergency department, to prompt a quick triage and evaluate the lung involvement [38].

**Figure 3:**
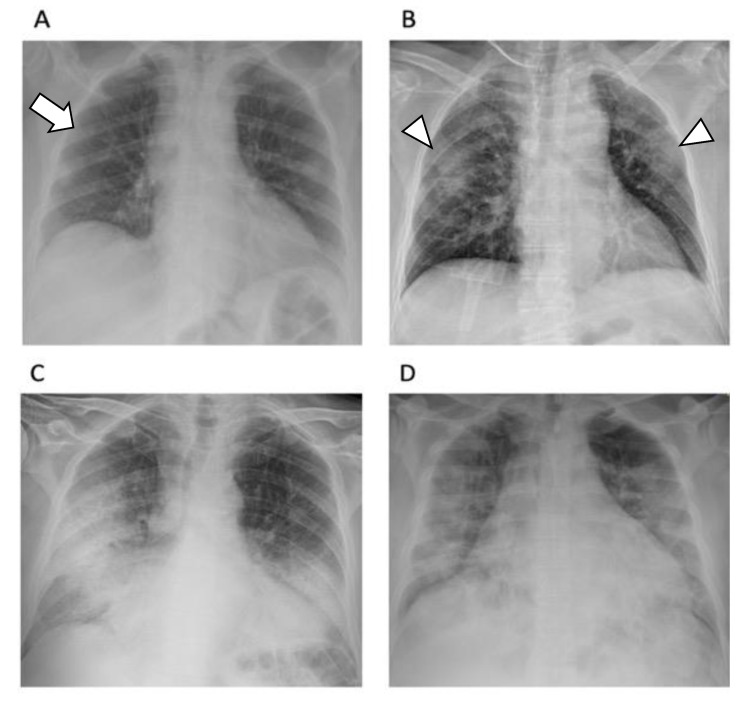
Chest X-ray of patients affected by SARS-CoV-2 according to the extent of lung abnormalities involvement. (**A**). Lung involvement ≤ 25% (arrow). (**B**). Lung involvement 25–50% (arrowheads). (**C**). Lung involvement 50–75%. (**D**). Lung involvement ≥ 75%.

The CXR severity score is associated with the risk of intubation and hospitalization time [39]. Moreover, together with clinical data, CXR may help in predicting mortality and the need for ventilatory support [40]. Based on the American College of Radiology recommendations [41], CXR should be considered as a first diagnostic tool during pandemic peaks to obtain a quick, cheap, safe, and reproducible evaluation of lung involvement (Figure 3), although diagnostic accuracy is limited.

### 2.7. The Usefulness of Lung Ultrasound (LUS)

Lung ultrasound is a non-invasive bedside technique used to diagnose interstitial lung syndrome through evaluation and quantitation of the number of B-lines, pleural irregularities and nodules or consolidations, especially useful in patients admitted to the intensive care unit [42]. Moreover, the presence of inhomogeneous bilateral pattern of multiple B-lines and white lung, with scattered areas, characterizes ARDS.

In patients with COVID-19 pneumonia, LUS can reveal a typical pattern of diffuse interstitial lung syndrome, characterized by multiple bilateral B-lines with spared areas, thickening and irregularity of the pleural line and, sometimes, peripheral consolidations. All these findings are similar to the abovementioned features typical of CT [43].

A recently published meta-analysis by Jari et al. [44], by including 16 eligible studies, demonstrated that the pooled sensitivity and specificity were 86.9% and 62.4%, respectively, compared with 93.5% and 72.6%, respectively, for CT. The authors demonstrated that LUS reported an acceptable sensitivity at the cost of low specificity in the diagnosis of SARS-CoV-2 lung involvement.

## 3. Cardiovascular Involvement

### 3.1. Cardiac Involvement

Cardiac involvement in SARS-CoV-2 infection has been reported and it is likely related to either direct or indirect damage [45]. The direct damage is related to the presence of the ACE2 receptor in cardiomyocytes [46,47], while the indirect one is probably caused by the systemic inflammatory response syndrome including cytokine storm, dysregulated immunocytes, and uncontrolled inflammation [25], driving endothelial cell dysregulation with the consequent pro-thrombotic phenotype [48], inducing acute coronary syndrome.

Although SARS-CoV-2 cardiac involvement is defined as an increase of troponin above the 99^th^ percentile of the upper reference limit during the disease, troponin levels are not accurate [49]. Therefore, myocardial involvement needs a global evaluation, including imaging in selected cases, based on the European Society of Cardiovascular Radiology (ESCR) recommendations [50].

The most common cardiac conditions occurring in COVID-19 patients are acute coronary syndrome (ACS) (4–25%) [51], myocarditis (8–12%), and cardiac dysrhythmia (6–17%) [6].

### 3.2. The Usefulness of Cardiac Imaging

As ESCR recommended, Cardiac Computed Tomography Angiography (CCTA) is the best choice to study patients with COVID-19 disease with suspicious cardiac involvement [50]. CCTA is preferred to transesophageal echocardiography in the evaluation of left atrial appendage thrombus in patients with arrhythmia or the study of valve endocarditis, for the safety of operators and patients [50]. CCTA may help for the evaluation of coronary arteries for excluding the presence of suspicious ACS, to avoid unnecessary angiographic catheterization [50].

Overall, CT is a valid tool to identify pneumonia, obstructive coronary artery disease, and pulmonary embolism, with the advantage of a triple rule out [6,50,51].

### 3.3. Peripheral Vascular Involvement

Vascular involvement by SARS-CoV-2 has been well established in recent literature. The biochemical mechanisms leading to endothelial attachment and subsequent inflammation and dysfunctions, often with thrombotic manifestations, have been investigated [7].

### 3.4. Peripheral Vein Thrombosis

Lower and upper extremity Doppler ultrasound is the first-line imaging modality for diagnosis of peripheral venous thrombosis [52]: it is rapid, cost-effective, and can be performed at the bedside. In a systematic review of observational studies reporting the incidence of venous or arterial thromboembolism by Boonyawat et al. [53] based on compression ultrasound (CUS) screening, COVID-19 patients had a high incidence of deep vein thrombosis (DVT), particularly if hospitalized in ICUs.

Another multicenter prospective study including 227 consecutive patients with moderate-severe COVID-19 pneumonia showed a relevant incidence of DVT in acutely ill patients with COVID-19 pneumonia, mostly asymptomatic [54]. Based on the results, these studies recommend a surveillance protocol by serial CUS of the lower limbs to timely identify DVT.

### 3.5. Peripheral Arterial Thrombosis

Cases of atypical presentation of COVID-19 with thrombosis at multiple sites, involving peripheral arteries in the upper or lower limbs have been reported [55]: the diagnosis was achieved through total-body CT angiography or CT angiography for upper or lower extremities. The retrospective study by Goldman et al. [56] witnessed an elevated positivity rate (100% patients had at least one lower extremity clot) amongst CT angiographic studies performed for claudication symptoms in COVID-19 patients with a higher clot burden and worse prognosis (higher incidence of amputation or death) in test population when compared with the control group. Considering the risk of thromboembolic events, low molecular weight heparin (LMWH) at prophylactic dose is considered as an option in bedridden patients with acute COVID-19 infection, as thrombotic processes in the vessels induced by the inflammatory cascade is described [57]. Post-thrombotic sequelae have been less widely considered in recent literature, as the long-term effects of the infection are not yet clear [58].

## 4. Central Nervous System Involvement

ACE2 receptor is expressed also in glial cells and neurons of the mammalian brain, in particular on the membrane of brainstem nuclei involved in cardiopulmonary function [59]. According to this evidence, SARS-CoV-2 is considered to have potential neuro-invasiveness that might lead to acute brain disorders within his replication and infection of CNS cells [60].

The hypothesis of COVID-19 neuro-invasion is confirmed by a growing incidence of a wide range of neurological manifestations [8]. During the various waves of the 2020 crisis, it was increasingly evident that SARS-CoV-2 can contribute to several neurological manifestations including anosmia, seizures, stroke, confusion, encephalopathy, and total paralysis [61].

The reference standard imaging technique for patients with suspected COVID-19 neurological involvement is brain MRI [62].

**Figure 4:**
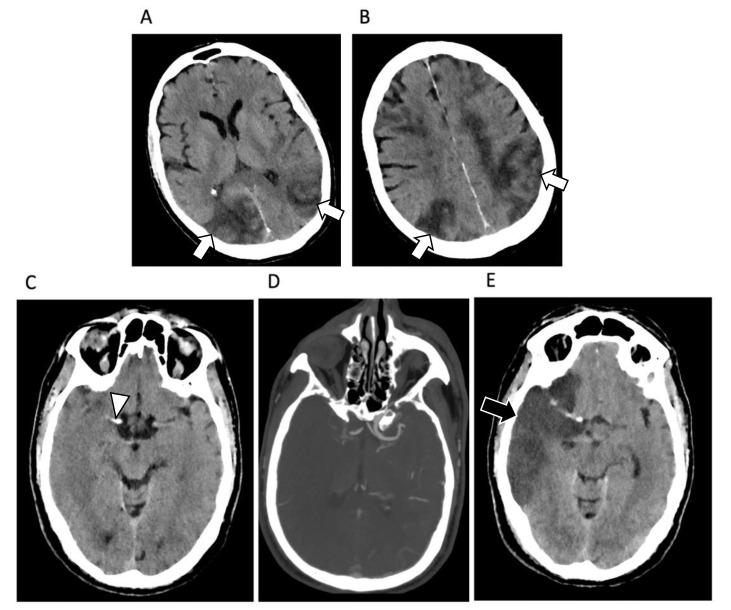
Brain findings in COVID-19 patients. (**A**,**B**) Axial brain CT images of a 56-year-old man affected by SARS-CoV-2-related pneumonia showing multiple hypoattenuating areas located in the cortical junction bilaterally, with a predominant extension in the parietal and occipital lobes, due to ischemia (white arrows). (**C**) Axial brain CT images of a 71-year-old woman affected by SARS-CoV-2-related pneumonia showing hyperdense artery sign in the right middle cerebral artery (white arrowhead), due to acute thrombosis. This finding was confirmed by brain CT angiography (**D**) showing a complete thrombosis of the middle cerebral artery. After 15 days brain CT images (**E**) show a large hypoattenuating area in the fronto-parietal lobe (black arrow) due to the brain ischemia.

Egbert et al. [63] collected data from 361 patients with COVID neurological symptoms and, in this court, brain abnormalities suggestive of COVID-19 etiology were present in 34%. The most common brain lesions were: white matter (WM) hyperintensities, microhemorrhages, infarct, edema, ischemia, hematoma, and smaller olfactory bulb (Figure 4).

WM hyperintensities, which together accounted for 76% of affected cases were bilateral. Changes were also registered in the insular cortex, cingulate gyri, cerebral peduncle and internal capsule and basal ganglia, splenium of the corpus callosum, olfactory nerves/bulb, and gyrus rectus or described as diffuse [64].

Microhemorrhages in WM were noted in about 13% with a bilateral diffuse presentation, in corpus callosum and putamen, bilateral juxtacortical WM, and internal capsule [64]. However, Dixon et al. [65] associate the presence of micro-bleeds in COVID-19 patients with severe hypoxia and not with SARS-CoV-2 neuro-invasion.

Brain stroke was reported in about 10% of cases, mainly with involvement of bilateral anterior and posterior circulation territories. Hemorrhages were noted in about 6% of cases with multiple and various locations, more commonly with bilateral involvement, usually of posterior parieto-occipital lobes and corpus callosum, frontal, occipital, and temporal areas including Sylvian fissure or with lateral ventricles involvement [66,67].

Nonspecific edema was reported in 3% of cases in bilateral WM with a diffuse presentation [68].

The likely association of COVID-19 with cerebrovascular disease may reflect an increased risk of secondary vessel thrombosis [69].

Gulko et al. [70], analyzed other 126 patients with brain MRI and classified neuro-COVID patients according to the most common interpretation of MRI abnormalities and not only on the type of lesions. The most commons diagnoses were acute or subacute infarcts (25.3%), posterior reversible encephalopathy syndrome (PRES), hemorrhagic lesions (3%), dural venous sinus thrombosis (1.5%), demyelinating lesions (2.3%), acute hemorrhagic necrotizing encephalopathy (0.07%), and hypoxic-ischemic encephalopathy (0.07%).

Even though brain radiological features in CNS involvement are still nonspecific and not strictly related to the clinical symptoms, literature underlines the importance of MRI to better evaluate SARS-CoV-2 brain infection [71].

## 5. Abdominal Organs Involvement

COVID-19 infection may cause involvement of abdominal parenchymal organs and the gastrointestinal (GI) tract. The causative mechanisms include direct viral infection, indirect thromboembolic complications, or indirect drug-induced complications [9,10,11].

Interestingly, patients with severe COVID-19 have significantly higher rates of abdominal pain [odds ratio (OR) = 7.10] compared with those with non-severe disease [72]. The occurrence of abdominal imaging manifestation in COVID-19 patients may be incidental in some cases and it may be difficult to understand if COVID-19 infection is the causative mechanism [73,74]. US should be the first imaging technique to assess the hepatobiliary system, the spleen and the genitourinary involvement, while abdominal x-ray may be used to exclude the suspicion of bowel obstruction or perforation. If ultrasound or x-ray are doubtful, abdominal CT should be then performed. In case of suspicion of vascular involvement of spleen, kidney or gastrointestinal which may lead to ischemia, abdominal CT should be performed as first imaging technique. The assessment of pancreas is doable but usually difficult on ultrasound, which means that in most cases abdominal CT is indicated; however, it is worth mentioning that in case of suspicion of pancreatitis, abdominal CT should be performed after 3 to 5 days after symptom onset to best assess if edematous or necrotic and complications.

### 5.1. Hepatobiliary System

Baseline laboratory investigations performed on the suspicion of COVID-19 infection at hospital admission usually include the assessment of hepatic function [5]. Abnormal liver function at hospital admission is encountered in more than one-third of patients with SARS-CoV-2 infection [75] and is associated with systemic inflammation, longer means hospital stays, organ dysfunction, and admission to ICU or even death [75,76].

A retrospective study including COVID-19 patients who performed abdominal imaging examinations demonstrated that about half of the patients had findings of gallbladder bile stasis, which is however common in critically ill patients [77]. Interestingly, less than 3% of patients who performed CT in this study had signs of liver injury [78]. There are several reports of acute acalculous cholecystitis and it is not clear whether this is associated directly with ACE2 receptor expression in the gallbladder wall or with prolonged parenteral nutrition [79,80].

### 5.2. Pancreas

Abnormality in amylase or lipase values has been encountered in about 12–17% of COVID-19 patients [81]. However, the causes of high levels of amylase or lipase may be not related to pancreatic injury itself [82], and rarely result in acute pancreatitis [83]. Pancreatic involvement in COVID-19 infection is theoretically likely due to the presence of ACE2 receptors in the pancreas [84].

Overall, about 3% of COVID-19 patients with abdominal CT scans show findings of pancreatitis [77]. Funt et al. [74] found a similar rate (about 11%) of pancreatitis on CT in COVID-19 and non-COVID-19 patients who underwent abdominal CT in the emergency department in the same timeframe. In a study by Dirweesh et al. [85] the occurrence of acute pancreatitis was similar in COVID-19 and non-COVID-19 patients, but patients with COVID-19 had a higher Charlson Comorbidity Index and Bedside Index of Severity in Acute Pancreatitis scores on presentation. Interestingly, in two studies comparing non-severe and severe COVID-19 patients, there were no laboratory or imaging signs of pancreatitis in patients within the former group while pancreatitis was detected in 7.5–32.5% of patients with severe COVID-19 disease [85,86].

### 5.3. Spleen

Baseline laboratory investigations performed on the suspicion of COVID-19 infection at hospital admission oftentimes show lymphocytopenia, thrombocytopenia, and leukopenia [87].

Splenic involvement in COVID-19 infection has been reported in a few cases at imaging. Imaging findings of splenic involvement in COVID-19 include spleen size increase in the early stages [88], splenic infarction [77,89] as well as atraumatic splenic rupture, and arterial thrombosis [90].

### 5.4. Gastrointestinal Tract

Digestive symptoms occur in about 15% of COVID-19 patients with nausea or vomiting, diarrhea, and loss of appetite being the three most common symptoms [72].

**Figure 5:**
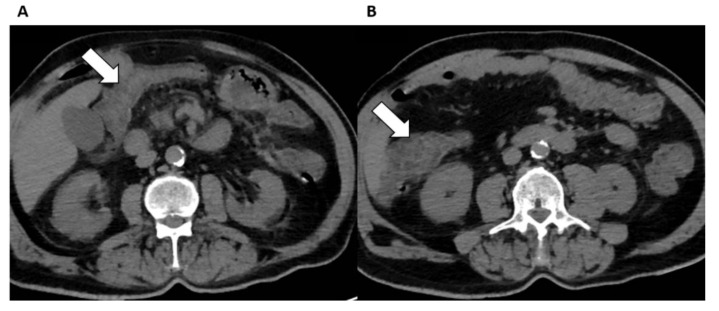
Intestinal findings in a COVID-19 patient admitted to the Emergency Department for abdominal pain. (**A**,**B**) Unenhanced axial abdominal CT images show bowel wall thickening in the transvers colon (arrows) with adjacent fat stranding consistent with intestinal involvement with ischemic lesions.

Abdominal CT scans performed in hospitalized patients with COVID-19 showed abnormalities in the bowel wall or fluid-filled colon in 18–31% and 43% of patients, respectively, with a significantly higher occurrence of these findings in ICU patients as compared to patients not in the ICU [78]. Bowel abnormalities (Figure 5) on CT include findings of inflammation, ischemia, obstruction, diverticulitis, and, even, perforation [74,78]. Macrovascular arterial or venous thrombosis is identified in almost half of COVID-19 patients with bowel ischemia [91]. Interestingly, abnormalities in the bowel seem to occur independently of severity of pulmonary involvement, other clinical and laboratory features [92].

## 6. Genitourinary Involvement

In the genitourinary system, the kidneys are the most affected organ in patients with COVID-19. Renal injury most commonly manifests with increased serum creatinine, proteinuria, hematuria, and low glomerular filtration rate. Renal insufficiency is a serious complication in critically ill patients with COVID-19 and it may occur at any time in the course of the disease [93]. Acute kidney injury has been reported in up to 16–55% of hospitalized COVID-19 patients and it has been strictly associated with the severity of the pulmonary disease and older patients’ age [94,95]. Moreover, impaired renal function is a poor prognostic factor in hospitalized patients, being associated with longer hospitalization length stay, high in-hospital mortality, and subsequent chronic kidney disease in survivors, with the need of a kidney replacement therapy [96,97].

The pathophysiological mechanisms of renal damage during COVID-19 infection are still debated. Several causes have been postulated including direct cellular injury due to the expression of ACE2 in tubular cells, inflammatory and immune over-reaction with glomerular cells injury, a pro-coagulant state with microvascular thrombosis, as well as secondary damage due to hemodynamic instability (i.e., systemic hypotension or reduced cardiac output) and nephrotoxic drugs in critically ill patients [98,99].

Involvement of other urogenital organs, such as the urinary bladder, is rare in COVID-19 patients, and bladder wall thickening or hemorrhage has been occasionally described [100].

### Kidneys

Radiologists play a crucial role in the early identification of renal damage in COVID-19 patients. US should be considered as the first-line modality at the time of admission in patients with impaired renal function and for renal imaging in critically ill patients, as it can be performed in the intensive care unit at the bedside and can be repeated for close patient’s follow-up. US provides a quick and non-invasive assessment of renal morphology and echogenicity, vascular patency of the main renal vessels, and renal resistive index on color Doppler to detect potential renal microcirculation damage. Increased echogenicity of the renal cortex and loss of corticomedullary differentiation can be observed in patients with acute kidney injury [100]. Moreover, ultrasound can rule out other causes of renal damage not related to COVID-19, such as obstructive nephropathy.

Abdominal CT may be performed at the time of admission for the initial workup of critically ill patients with abdominal symptoms. On unenhanced CT images, decreased parenchymal density and perinephric fat stranding were associated with impaired renal function in COVID-19 patients [101].

Radiologists’ evaluation of kidneys should pay particular attention to the patency of main renal vessels due to the known risk of thromboembolic complications in COVID-19 [10]. The kidney is the second most common site of abdominal ischemia after the bowel, with a reported incidence of renal infarct of 3–19% in patients undergoing contrast-enhanced CT. Renal artery thrombosis can be secondary to embolic phenomena or due to local thrombus development. Contrast-enhanced CT findings include occlusion of the renal artery or its branches with ipsilateral parenchymal infarct characterized by wedge shape hypointensity of the renal cortex [102]. Global renal infarct can occur in case of complete thrombosis of the main renal artery, while bilateral infarcts have been occasionally described in patients with COVID-19 [102]. Renal vein thrombosis has also been reported in COVID-19 patients with concomitant pulmonary embolism [102]. Other renal complications such as pyelonephritis are less commonly observed in COVID-19 patients [103].

## 7. Conclusions

Although COVID-19 is reported as a disease that primarily affects the lungs, it can involve and damage several other organs, increasing the risk of both acute and long-term health problems. Moreover, even if clinical manifestations of SARS-CoV-2 are nowadays mild in comparison to the first wave, it is important to remember all the different and ambiguous clinical scenarios that we can face in everyday practice, due to the wide expression of ACE2 receptor. For this reason, it is paramount that radiologists increase the awareness of SARS-CoV-2 whole-body involvement, to help solve the differential diagnosis, quickly identify possible complications, and guarantee the best management possible.

## Figures and Tables

**Table 1:** Proposed CT classification for patients with suspected involvement by SARS-CoV-2.

CT Appearance	Rationale	Findings
**Typical**	Common imaging features of greater specificity for COVID-19 pneumonia	Peripheral, bilateral, GGO with or without consolidation or crazy paving Multifocal GGO of rounded morphology with or without consolidation or crazy paving Reverse halo sign or other findings of organizing pneumonia
**Indeterminate**	Nonspecific imaging features for COVID-19 pneumonia	Absence of typical features AND presence of: Multifocal, diffuse, perihilar, or unilateral GGO with or without consolidation lacking a specific distribution and nonrounded or no peripheral Few very small GGO with a nonrounded and no peripheral distribution
**Atypical**	Uncommon imaging features for COVID-19 pneumonia	Absence of typical or indeterminate features AND presence of: Isolated lobar or segmental consolidation without GGO Discrete small nodules (centrilobular or “tree-in-bud”) Lung cavitation Smooth interlobular septal thickening with pleural effusion
**Negative for pneumonia**	No features of pneumonia	No CT features to suggest pneumonia

Adapted from Simpson S. et al. [33], (2020) Radiological Society of North America Expert Consensus Document on Reporting Chest CT Findings Related to COVID-19: endorsed by the Society of Thoracic Radiology, the American College of Radiology, and RSNA. Radiology: Cardiothoracic Imaging 2:e200152, doi:10.1148/ryct.2020200152. GGO: ground-glass opacities.

## Data Availability

Not applicable.
